# Development and characterization of a pentylenetetrazol-induced convulsive seizure model in non-anaesthetized sheep

**DOI:** 10.1093/biomethods/bpaf086

**Published:** 2025-12-01

**Authors:** Ruslan V Pustovit, Yugeesh R Lankadeva, Ming S Soh, Sam F Berkovic, Christopher A Reid, Clive N May

**Affiliations:** Preclinical Critical Care Unit, Florey Institute of Neuroscience and Mental Health, University of Melbourne, 30 Royal Parade, Parkville, VIC 3052, Australia; Preclinical Critical Care Unit, Florey Institute of Neuroscience and Mental Health, University of Melbourne, 30 Royal Parade, Parkville, VIC 3052, Australia; Epilepsy Group, The Florey Institute of Neuroscience and Mental Health, University of Melbourne, 30 Royal Parade, Parkville, VIC 3052, Australia; Epilepsy Research Centre, Department of Medicine, Austin Health, University of Melbourne, 145 Studley Rd, Heidelberg, VIC 3084, Australia; Epilepsy Group, The Florey Institute of Neuroscience and Mental Health, University of Melbourne, 30 Royal Parade, Parkville, VIC 3052, Australia; Preclinical Critical Care Unit, Florey Institute of Neuroscience and Mental Health, University of Melbourne, 30 Royal Parade, Parkville, VIC 3052, Australia

**Keywords:** seizure, animal model, sheep, PTZ, epilepsy, SUDEP, cardiovascular system

## Abstract

The pathophysiology of seizures is complex and could contribute to a range of morbidities including sudden unexpected death of epilepsy (SUDEP). A better understanding of seizure-induced pathophysiology can lead to the development of targeted interventions. Here, we describe the development and characterization of a novel large mammalian model of convulsive seizures in non-anesthetized sheep induced by pentylenetetrazol (PTZ), one of the most widely used proconvulsant drugs in epilepsy research. A dose of intravenous PTZ that reliably induced a reproducible and consistent level of seizure in non-anaesthetized sheep was determined. Convulsive seizures went through a relatively predictable sequence, similar to that seen in other animal models of epilepsy. A species-specific seizure severity scale system, based on the field Racine’s scale that is widely used in epilepsy research, was designed to establish a user-friendly scoring system for PTZ-induced seizures in sheep. We demonstrated that convulsive seizures caused substantial increases in mean arterial pressure and heart rate. The translational value of this large animal model can be further enhanced when combined with other translational tools such as quantitative systems physiology and pharmacology, potential biomarker testing and experimental preclinical trials of potential prophylactic treatments. An advanced animal model, such as described in this study, provides a unique opportunity for comprehensive physiological monitoring of neural and systemic pathways activated by interictal and ictal activity and can contribute to the development of preventive therapies for seizures.

## Introduction

Epilepsy is one of the most common serious chronic neurological diseases, affecting almost 70 million people worldwide [1]. Seizures induce multiple cardiovascular and respiratory changes that can lead to sudden unexpected death in epilepsy (SUDEP), which is the leading cause of death in patients with epilepsy. Several pathophysiological mechanisms have been proposed to explain SUDEP; however, the mechanisms of SUDEP remain largely unresolved and there are no prophylactic treatments [2, 3].

The frequency of recurrent generalized tonic–clonic seizures is the principal risk factor for SUDEP [4]. Yet, the unpredictable and complex changes in cardiovascular, respiratory, and autonomic function through which an ictal event may trigger a terminal outcome remain unclear [5]. It is impossible to study the mechanisms and treatment of SUDEP in humans; thus, an animal model is essential to investigate the factors leading to a terminal event in a controlled, well-monitored manner in a detail not possible in patients.

For the last 40 years, animal models that resemble human seizures have been the foundation on which numerous novel antiseizure drugs and potential mechanisms of SUDEP have been discovered [6]. The GABA(A) antagonist pentylenetetrazol (PTZ), which induces neuronal hyperexcitability, has been shown to induce proconvulsant activity in mice, rats, monkeys, and humans [7]. Simple seizure tests in rodents, such as PTZ administration and maximal electroshock stimulation, have been used extensively by the pharmaceutical industry and have been instrumental in identifying most of the currently clinically used antiepileptic drugs [6, 8, 9].

Rodent models of seizure have been widely studied, but, over the past few decades, sheep have gained prominence as models for replicating neurological disorders in humans [10, 11]. A sheep model of seizure offers several attractive traits compared with rodents. In particular, the sheep brain is gyrencephalic and shares a high degree of homology in size and structure with the human brain, which is advantageous for translational neurological research [11]. Furthermore, due to their size, it is possible to surgically instrument sheep with multiple sensors. This allows simultaneous monitoring of the electroencephalogram (EEG) together with arterial pressure, cardiac output, sympathetic nerve activity, measures of vagal nerve activity, and blood gases as a measure of pulmonary function. Such detailed measures are not possible in smaller animals. In addition, sheep being reasonably docile animals, in vivo monitoring can be performed in non-anaesthetized conditions to establish the pathophysiological factors related to the occurrence of SUDEP. There is a strong rationale for the development of a consistent and reproducible large mammalian model of seizures to understand the pathophysiology and establish an experimental testbed to assess the safety and efficacy of new therapies prior to clinical translation [3, 9, 12].

The goal of the current study was to establish a dose of PTZ that reliably induced reproducible convulsive seizures and EEG changes in non-anaesthetized sheep. Furthermore, we aimed to develop a modified severity scoring system to assess the intensity of PTZ-induced seizures. Together, these can be used to increase our understanding of the pathophysiology of seizures and to subsequently test the safety and efficacy of new interventions.

## Materials and methods

All experimental protocols and procedures were approved by the Animal Ethics Committee of the Florey Institute of Neuroscience and Mental Health (ID # 22-074) under guidelines laid down by the National Health and Medical Research Council of Australia and conformed with the ARRIVE and ARRIVE 2.0 guidelines [13].

Experiments were conducted on 10 healthy adult Merino ewes (37.7 ± 1.2 kg) housed in individual metabolic cages and fed 800 g of oaten/lucerne chaff per day and given water *ad libitum*. All animals were acclimatized to the laboratory environment (12-h light/12-h dark cycle) for at least 7 days prior to being moved to metabolic cages for experimentation.

## Surgery

Sheep were instrumented in an aseptic surgical procedure under general anaesthesia induced with sodium thiopentone (15 mg/kg; Jurox Pty Limited, Rutherford, NSW, Australia) and maintained with isoflurane (2.0%–2.5%, Isoflurane; Avet Health LTD, Lane Cove, NSW, Australia) in an oxygen/air/isoflurane mixture. For any surgical procedure, each sheep received intramuscular injections of antibiotic (15 mg/kg procaine penicillin, Ilium Propercillin; Troy Laboratories, Glendenning, NSW, Australia) and analgesic (2 mg/kg flunixin meglumine, Ilium Flunixil, Troy Laboratories, Glendenning, NSW, Australia) both pre- and post-operatively every 24 h for 3 days.

An electrode array to record the EEG was implanted. After the sheep was positioned in a stereotaxic frame, an incision of 4–5 cm was made along the scalp midline, with the caudal end of the incision at the level of lambda. The periosteum was removed, and a craniotomy 1.0 × 1.0 cm was made 1.0 cm lateral to the sagittal suture and 1.0 cm rostral to lambda to expose the dura mater. After the dura mater was incised, an electrode array (4 contact, sub-dural platinum electrode array; TS04R-SP10X-000, Medical Instrument Corporation, WI, USA) was inserted subdurally over the frontal cortex. The craniotomy was filled with haemostatic sponge cubes (SMI-SPON, SMI, Belgium) and protected with self-polymerizing dental acrylic (Vertex-Dental B.V., Soesterberg, The Netherlands). The cable was tunnelled under the skin to the back of the neck where it was exposed. One ground/reference electrode was placed over the skull under the skin. At the end of the surgical procedure, while sheep were still under general anaesthesia, a jugular vein was cannulated with two cannulas. In a subset of the sheep (*n* = 5), a carotid arterial loop was constructed [14]. After 2–3 weeks recovery, the carotid artery loop and a jugular vein were cannulated to measure mean arterial pressure and heart rate, and for drug administration, respectively. The patency of the cannulas was maintained by continuous infusion of heparinized saline (10 IU/mL at 3.0 mL/h) from a pressurized bag [14].

There were no surgical or postoperative complications in any sheep. The sheep were kept in individual cages for 1 week’s post-operative recovery before the start of experimental recordings.

## Induction of acute seizures with PTZ

All experiments were conducted on standing non-anaesthetized, unrestrained sheep in their metabolic cages. All experimental recordings were conducted between 9.00 a.m. and 01.00 p.m. to avoid any circadian-related behavioural variabilities between individual recordings. On the day of experiment, animals were connected to the recording equipment.

Three sheep were used for a dose response study in which PTZ (Pentylenetetrazol; Sigma-Aldrich, St Louis, MO, USA) dissolved in sterile 0.9% sodium chloride (Baxter, NSW, Australia) was administered intravenously at doses of 1.0, 3.0, and 5.0 mg/kg/min over 4 min. A dose of 5 mg/kg/min was selected for subsequent studies as it reliably induced a moderate level of seizure severity with development of convulsive seizures. Following cessation of seizure, experimental recordings were conducted for 60–90 min. One day after the experiment, animals were humanely euthanized with a lethal intravenous dose of sodium pentobarbitone (150 mg/kg; Lethaton, Randlab, Chipping Norton, NSW, Australia).

## Assessment of the effects of PTZ with video and EEG recording

For evaluation of seizures, animals were monitored both electrographically and behaviourally. All recordings took place between 9.00 a.m. and 01.00 p.m. and the mean duration of a session was about 3 h. The experiments were done in a bright-lit room, which facilitated high quality video recordings.

After 7–10 days of post-operative recovery, video EEG commenced with 90–120 min of baseline recording. A video camera was focused on the front of the cage, ensuring that the sheep was recorded frontally at all times. The video camera was connected to a computer, which recorded the video in real time. The sheep was connected to recording leads for EEG recording in an unrestrained state. The first 1 h served as a habituation period for the animals to acclimatize to the experimental conditions. During the final 30–60 min, the baseline behaviour was scored, and EEG was recorded to ensure that the animals were free of epileptiform activity prior to seizure induction. Acute seizures were then induced by intravenous infusion of PTZ (5 mg/kg/min over 4 min), followed by behavioural and electrographic evaluation of seizure activity. Analog signals of electroencephalography (EEG) were recorded on a computer using a CED micro 1401 interface and Spike 2 software (Cambridge Electronic Design, Cambridge, UK). The signal was sampled at 250 Hz, amplified 20,000×, and filtered at 0.1 to 1.0 Hz with a 50 Hz Notch filter. Post-PTZ, observations continued for a minimum of 6 min and were terminated when both behaviour and EEG activity had normalized for at least 15 min. To enable later synchronization of the video and EEG recordings, a digital stopwatch was placed directly in the picture frame of the camera.

## Seizure classification

The video and EEG recordings were analysed by two independent observers. The incidence and duration of different behaviours were recorded using a stopwatch and were based on notes taken by an experimenter during recordings and from the videos. Behavioural expressions that accompanied epileptic EEG abnormalities defined as patterns showing isolated spikes or spiking activity of minimally 1 s duration and an amplitude of at least twice the baseline EEG [15]. Afterwards, these data were compared with the Racine score according to Lüttjohann et al. [15]. An adapted seizure severity scale was designed that included specific sheep clinical signs, such as looking lost or confused, head nodding, and kneeling down (Table 1). When multiple different classes of severity occurred during one electrographically defined seizure, the most severe behaviour was used to score the severity of that seizure [16].

**Table 1.** Seizure severity scoring system: characterization of behavioural and EEG changes during an acute PTZ-induced convulsive seizure in non-anaesthetized sheep.

Score	Behaviour	EEG
0	No change in behaviour	Baseline
0.5	Animal looks lost and confused	Minor changes in amplitude activity
1	Extensive lip/tongue movement	Rising amplitude activity, isolated spikes
1.5	Ear twitching, stepping from limb to limb	High amplitude activity/slow waves
2	Head nodding	Prominent single high amplitude spikes
2.5	Head nodding, short-term myoclonic reflexes in the body	Poly high amplitude spikes and sharp waves
3	Forelimb clonus with kneeling	Spike bursts/spike and wave discharges
3.5	Forelimb clonus with kneeling, myoclonic reflexes in the body	
4	Forelimb clonus with lying and myoclonic seizures	Major spike bursts/spike and wave discharges
4.5	Generalized extensive myoclonic seizure	
5	Generalized tonic–clonic seizure	High intensity major spike bursts and wave discharges
5.5	Prolonged generalized tonic–clonic activity, unconsciousness	No information
6	Status Epilepticus (SE) and death	No information

## Data analysis

Analog signals of mean arterial pressure (MAP) and heart rate (HR) were recorded on a computer using a CED micro 1401 interface and Spike 2 software (Cambridge Electronic Design, Cambridge, UK) [17].

## Statistical analysis

All data were normally distributed as assessed by the Shapiro–Wilk test and are presented as means ± standard error of the mean (SEM). The number of observations for each variable is shown in the respective figure legends. Comparisons between baseline and predefined time points were analysed using one-way repeated measures ANOVA with a Bonferroni test to account for multiple comparisons. One-tailed *P *≤ 0.05 was considered statistically significant. Statistical analyses and figures were generated using GraphPad Prism Software (version 10.0, GraphPad Inc., CA, USA).

## Results

In this study, we characterized the PTZ model of acute convulsive seizures in non-anaesthetized sheep and have developed an evaluation scoring system based on the field Racine’s scale widely used in epilepsy research [18].

### Behavioural changes and seizure incidence induced by infusion of PTZ

In all, 10 sheep were successfully instrumented with EEG electrodes and allowed to recover for 7–10 days. Intravenous PTZ (5.0 mg/kg/min over 4 min) significantly changed behaviour. The natural behaviour diminished within 1–2 min after the PTZ infusion started and was replaced by early convulsive behaviours such as motionless and confused staring, intense tongue automatisms and/or ‘mouth cleaning-like’ behaviour, head nodding, and kneeling of animals. These substantial early changes in natural sheep behaviour may be considered as signs of seizure onset (Table 1).

**Figure 1:**
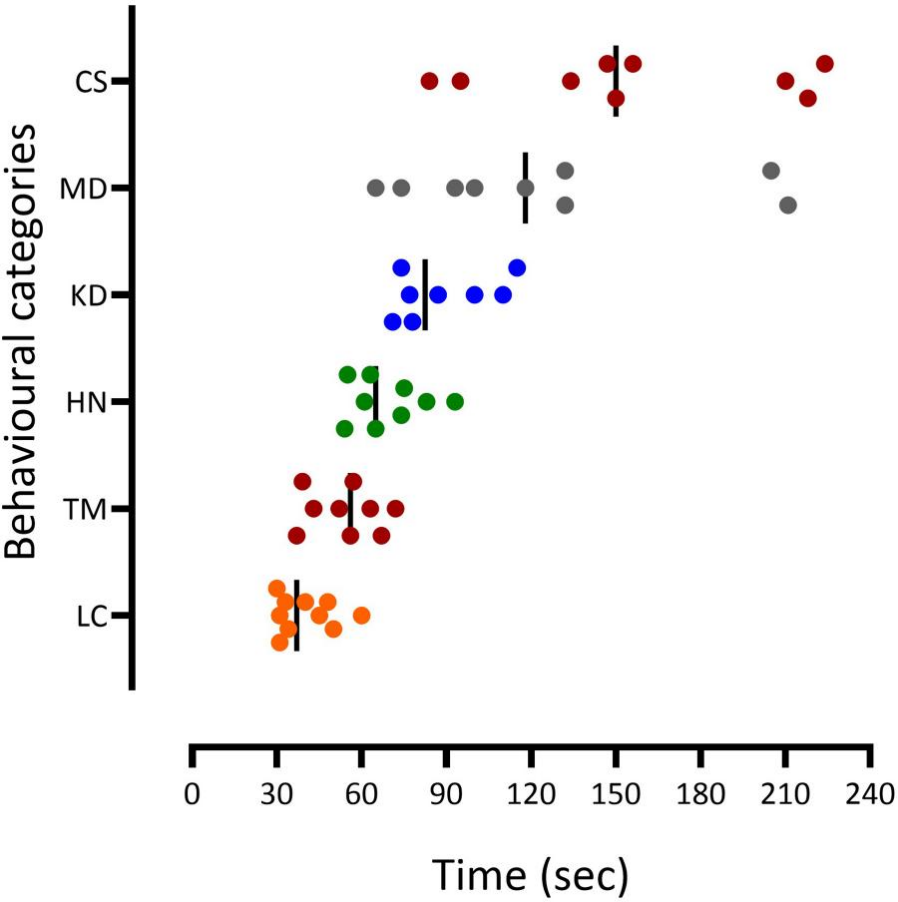
Time-related development of behavioural changes induced by intravenous PTZ (5 mg/kg/min from 0 to 240 s) in non-anaesthetized sheep. *Y* axis indicates behavioural categories: LC—lost/confused (orange), TM—extensive tongue/lips movement (dark red), HN—extensive head nodding (green), KD—kneeling down in a cage (blue), MD—muscle dystonia (grey), CS—convulsive seizure (maroon). Vertical lines indicate mean values

All of these early convulsive behaviours were observed at a similar latency from the start of the PTZ infusion (Fig. 1). After 40 ± 3 s (*n* = 10), all animals looked confused or lost (Score 0.5, Table 1). Sheep were less responsive to external stimuli by rapidly turning their heads in different directions in attempts to obtain more information, potentially feeling insecure in their usually safe environment. Unusual intense tongue movements or ‘mouth cleaning-like’ behaviours (Score 1.0, Table 1) were then observed at 54 ± 4 s after PTZ infusion started. Intense head nodding with pronounced ear twitching (Score 1.5–2.0, Table 1) occurred at 69 ± 4 s, which was observed simultaneously with hoof stamping to different degrees. Seven out of 10 experimental animals kneeled down (Score 2.5–3.0, Table 1) in their cages at 89 ± 6 s, and only three sheep remained standing until the first visible muscle convulsions were observed. Nine out of ten animals in our study developed these early convulsive behaviours in a similar sequence. Dystonia, a movement disorder presenting with sustained contractions of both extensor and flexor muscles, stretching movements and/or limb extension (Score 3.5–4.0, Table 1), was observed at 126 ± 17 s from the start of PTZ administration. By this stage, 7 out of 10 animals were lying in their cage, and the other 3 animals lay down from the standing position. One sheep skipped these stages and developed clear dystonia immediately after the signs of confusion were observed.

The most significant behavioural changes, development of generalized clonic seizures (GCSs), score 4.0–4.5 based on our modified seizure scoring system (Table 1), were observed over the first 4-min period after PTZ administration started. The average latency to GCSs (scores 4.0–4.5) was 157 ± 17 s, with each seizure episode lasting 61 ± 5 s. GCSs involved intensive and rhythmic simultaneous twitching and jerking of head, neck, body, and limb skeletal muscles. Most experimental animals developed only a single GCS, and only 3 out of 10 sheep developed secondary convulsive seizure after the PTZ intravenous infusion was stopped. Three out of 10 experimental animals developed a severe seizure score (5.0). In these sheep, generalized tonic–clonic seizures were observed with clear development of stiffening skeletal neck muscles. The duration of the tonic phase was 18, 23, and 25 s. No sheep reached the seizure severity score above 5.0, so data are not provided for these stages (Table 1).

Animals started to resume normal behaviour at 20–25 min post-seizure. This recovery was usually marked by long periods of immobility with sheep remaining in one fixed position and rarely displaying other behaviours. Sheep returned to their normal physiological behaviour at 37 ± 3 min, and by 60 minutes post-seizure, there was no discernible difference between baseline and postictal behaviour.

### Changes in EEG induced by infusion of PTZ

**Figure 2:**
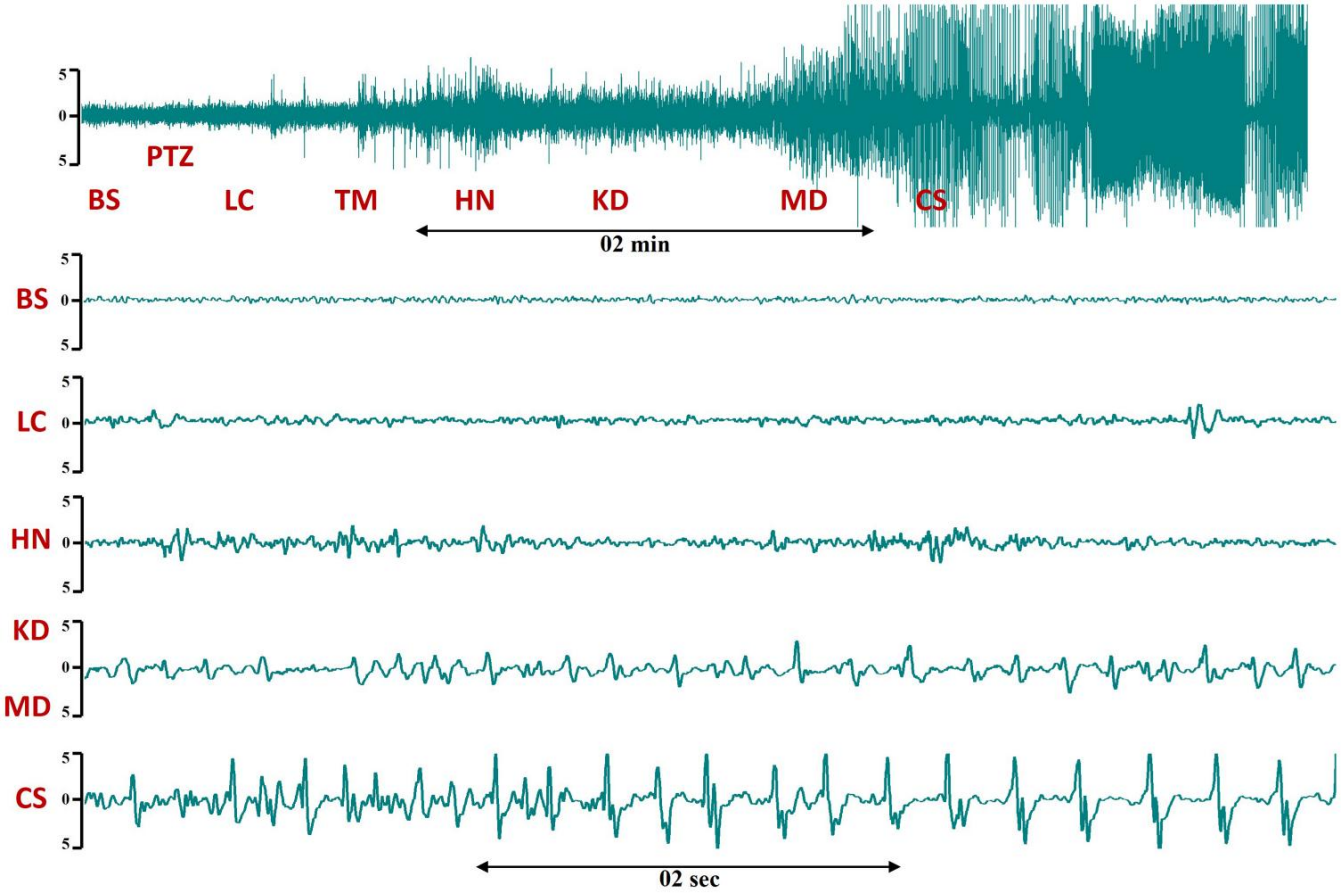
EEG traces during different behavioural categories of convulsive seizure induced by intravenous PTZ (5 mg/kg/min over 4 min) in non-anaesthetized sheep. Labelling of behavioural categories: BS—Baseline, pre-PTZ, LC—lost/confused, TM—extensive tongue/lips movement, HN—extensive head nodding, KD—kneeling down in a cage, MD—muscle dystonia, CS—convulsive seizure

Behavioural seizures were always associated with changes in EEG, with discharges easily detectable above background noise before the start of myoclonic seizures when the EEG often became contaminated by large amplitude electromyogram spikes. Figure 2 shows a typical intracranial EEG recording of a spike-and-wave discharge in a non-anaesthetized sheep with a PTZ-induced convulsive seizure. The state of appearing lost and confused in sheep can be characterized on EEG by a minor increase in EEG amplitude and activity (Fig. 2, LC). Isolated sharp spikes and slow waves were highly correlated with early convulsive behaviours like intense tongue movements or ‘mouth cleaning–like’ behaviour (Fig. 2, LC and TM). Prominent single, high-amplitude spikes and sharp waves were always accompanied by intense head nodding (Fig. 2, HN). Kneeling down and development of dystonia always coincided with spike bursts and wave discharges (Fig. 2, KD and MD). High-amplitude spikes, polyspikes, and major spike bursts with prolonged wave discharges accompanied the development of all generalized convulsive myoclonic seizures in sheep (Fig. 2, CS). In these epileptic bursts, it was sometimes observed that an increase in frequency or amplitude coincided with a transition of a clonic seizure to a tonic–clonic seizure.

### Changes in systemic haemodynamics induced by infusion of PTZ

**Figure 3:**
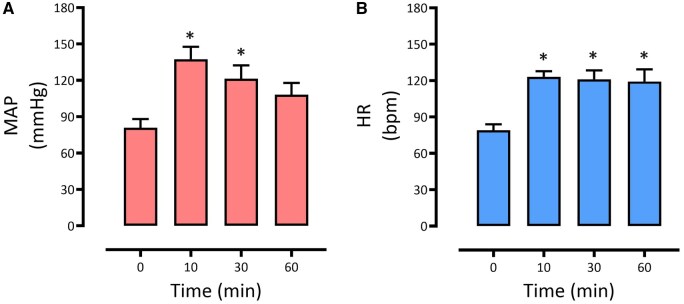
Changes in mean arterial pressure (A) and heart rate (B) induced by convulsive seizures in non-anesthetized sheep (*n* = 5). Values are presented as mean ± SEM. *P*-values are outcomes of one-way ANOVA with multiple comparisons. *P < 0.05

MAP rapidly increased from the baseline level of 80.9 ± 7.0 mmHg to 137.4 ± 10.3 mmHg (*P* = 0.0038) at 10 min post-seizure (Fig. 3). From this point, MAP gradually declined to 121.4 ± 10.9 mmHg at 30 min post-seizure, which was significantly higher than the baseline level (*P* = 0.0404), and to 108.2 ± 9.7 mmHg (*P* = 0.2287) at 60 min post-seizure. Similarly, there was a rapid increase in HR at 10 min post-seizure from 79.1 ± 4.8 to 123.1 ± 4.6 beats/min (*P* = 0.0021), which remained significantly increased for 60 min (119.2 ± 9.9 beats/min, *P* = 0.0046).

## Discussion

The aim of this study was to develop and characterize a large-animal model of reliably induced consistent and reproducible seizures. We elected to use sheep because of their large size, more similar anatomy and physiology to humans than rodents, docile nature, ready availability, and economic advantage over primates. We used PTZ as a convulsant as it has been shown to reliably induce proconvulsant activity in many species including humans. We determined a dose of PTZ that reliably triggers convulsant seizures in sheep and showed that this dose produced marked changes in the behavioural repertoire of these animals, accompanied by changes in EEG. We characterized the stages of convulsive seizure development and designed a modified severity scoring system based on Racine’s scale, the most widely used scoring system in epilepsy research. The PTZ-induced convulsive seizures led to rapid increases in MAP and HR, which remained substantially elevated above the baseline level up to 60 min post-seizure. To our knowledge, this is the first characterization of the ability of PTZ to provoke acute motor seizures in sheep.

### Animal models of seizure

Due to the significant problem of SUDEP, numerous preclinical studies have investigated the systemic consequences of seizure activity on cardiorespiratory function. Rats and mice have been typically used as induced seizure models to study the pathophysiological effects of seizures that may contribute to SUDEP [5]. As the seizure onset can be controlled in acute models, it is possible to predict and plan the time for physiological monitoring of the terminal seizure and application of targeted candidate preventative treatments.

Maximal electroshock stimulation has been widely used to induce a SUDEP-like event in adult mice, with mortality up to 62.5%. Kainic acid injection into the ventral hippocampus in rats is associated with prolonged convulsive seizures; however, tonic–clonic seizures, the biggest risk factor for SUDEP, are extremely rare even after high doses of kainic acid [7]. Bicuculline reliably induced seizures after systemic administration, although it has significant disadvantages. Bicuculline is very difficult to dissolve, is unstable, and lacks an exact mechanism of action as it is not a pure GABA-A receptor antagonist [19]. In anaesthetized hemispherectomized rats, topical penicillin activated hypothalamic and mesencephalic cardioarrhythmic triggers that induced spontaneous vagal nerve activity that could lead to lethal cardiorespiratory and metabolic changes [20]. These models provide a reliable induction of motor seizures and have helped to shed light on certain pathogenic mechanisms of SUDEP. These studies have led to hypotheses that seizure spread into the cardiorespiratory regions of the brainstem is central to the development of SUDEP [21–24]. However, the translatability of these and other models is very limited, as often animals were anaesthetized during experiments [2, 4, 12, 20, 25, 26].

For more than 50 years, PTZ has been used successfully to elicit seizures in rats [7], mice [7, 16], piglets [4], nonhuman primates [9], dogs [2], and cats [26]. These preclinical models have been used for the development of many of the antiseizure drugs that are clinically used today [6]. However, to our knowledge, PTZ has not been used in sheep. Our study is the first to demonstrate that systemic administration of PTZ reliably elicits seizures in non-anaesthetized sheep and induces all four behavioural seizure phenomena: freezing, myoclonic twitches, clonic, and tonic–clonic seizures. The seizures were reproducible and went through a relatively predictable sequence that is seen in other animal models of epilepsy.

### Advantages of sheep as animal model of seizure

The past two decades have seen a considerable rise in the use of sheep to model human neurological disorders. The development of an acute PTZ model of seizure in sheep is logical and clinically relevant because of the phylogenetic proximity of sheep to humans. In particular, sheep have a brain and heart more comparable in size and structure to humans than rodents [9]. Sheep are relatively cheap and easy to house compared with other large laboratory animals. In addition, sheep are more outbred compared with laboratory rodents, thus closely reflecting the heterogeneity of the human population, particularly when it comes to disease pathophysiology. Being reasonably docile animals, sheep are easy to work with and can be assessed using a wide range of in vivo monitoring techniques. Importantly, experiments can be performed on non-anaesthetized, unrestrained sheep avoiding the confounding effects of general anaesthesia. Sheep are amenable to regular sampling of blood and cerebrospinal fluid, aiding biomarker discovery and monitoring of treatment safety and efficacy. Moreover, compared with nonhuman primates, sheep are cost-effective alternatives and easier to manage in an experimental setting. Increasingly, novel human therapeutics are being developed in sheep models of disease and are receiving United States Food and Drug Administration (FDA) clearance for clinical translation [3, 11]. A further important advantage of sheep is that it is possible to make multiple simultaneous physiological measurements in non-anesthetized sheep that will provide information on the possible mechanisms by which seizures induce SUDEP. Such measurements include EEG together with arterial pressure, cardiac output, cardiac and renal nerve activity, and measures of vagal tone [27–29].

In a sheep model of status epilepticus (SE), induced in non-anesthetized sheep by intravenous administration of bicuculline, central apnea and cardiac abnormalities were suggested to contribute to SUDEP [30, 31]. However, this sheep model of seizure is limited by the use of bicuculline, which promotes hyperventilation in kittens, rats, and piglets. In a similar model, the aetiology of the pulmonary oedema was proposed to result from pulmonary hypertension due to centrally induced apnoea [32]. In these pioneering preclinical studies, death occurred after prolonged generalized tonic–clonic seizures that caused massive derangements of metabolism, cellular energetics, ion homeostasis, and systemic physiology, whereas in patients, SUDEP occurs after short convulsive seizures that by themselves would not be expected to be fatal [11, 19].

Opdam and colleagues were the first to show that penicillin administration to the frontal cortex of anaesthetized sheep could elicit focal seizures with secondary spread [12]. This model allowed for concurrent EEG and fMRI data collection and was instrumental in deepening the understanding of seizure generation and spread in sheep. However, these studies were conducted in anaesthetized, immobilized sheep and cortical administration of penicillin required invasive procedures. Our model of seizure is in non-anaesthetized sheep and has the advantage that a well-established proconvulsive compound is administered by a simple intravenous infusion, resulting in reproducible and consistent convulsive seizures in all sheep.

### Scoring scale

Seizure severity in experimental models of epilepsy is often evaluated by means of the Racine scale, in spite of the use of seizure induction methods that are different from those in the original paper by Racine in 1972 [15, 18]. It is, however, questionable whether use of the Racine scale for the assessment of seizure intensities in other epilepsy or seizure models is justifiable. The Racine scale has been applied to the PTZ seizure model in mice [16] and many of the behavioural expressions of seizure in rats and mice occurred in sheep. Since certain behavioural and EEG seizure characteristics are distinct between species, we designed a species-specific seizure severity scale to establish a user-friendly scoring system for PTZ-induced seizures in sheep.

We identified clear behavioural differences between the seizure stages in sheep that we clustered into 12 different categories (Table 1). This new species-modified seizure severity scoring system precisely describes the behavioural changes observed during different stages of PTZ-induced seizures, making it easier to assess the state of the animal and detect the first signs of convulsive seizures early and precisely. Furthermore, the improvement in inter-observer reliability should lead to a smaller variance in the data, so that less animals are needed in future studies.

### EEG changes

Classically, the characterization of an animal model of seizure requires evidence that the observed paroxysmal behaviour is epileptic in origin, using simultaneous EEG recordings, preferably through video EEG (vEEG). Despite certain limitations, EEG and vEEG studies are considered the gold standard in clinical practice to diagnose epileptic seizures [33]. Synchronicity of video and EEG recordings is critical, because normal behaviours of animals (drinking, chewing, stamping, etc.) may produce some artifacts or alternatively may generate state-related changes in the EEG. In our model, we simultaneously recorded intracranial EEG as a measure of changes in brain electrical activity together with changes in behaviour using a synchronized external video camera.

### Systemic haemodynamic changes

Early studies in anesthetized sheep models of seizure, induced by intravenous administration of bicuculline, demonstrated increases in systemic arterial pressure [34, 35]. Similarly, in the present study, we demonstrated that PTZ-induced seizures were accompanied by rapid, large increases in MAP, which remained substantially elevated for 60 min post-seizure. Seizures also caused a significant tachycardia despite the increase in MAP, likely due to the increase in cardiac sympathetic activity and decrease in vagal tone. Importantly, the experiments in our study were completed in the absence of anaesthesia. These seizure-induced changes in autonomic balance, together with the increase in MAP and tachycardia, can potentially promote the development of cardiac arrhythmias, which have been postulated as one of the major contributors to the development of SUDEP [36].

## Conclusions

Preclinical models are essential to understand the pathophysiology of human diseases that involve multiple organ systems and to design and test new treatments. We have developed a non-anaesthetized sheep model in which PTZ reliably induced seizures that went through a predictable sequence seen in other animal models of epilepsy and in epileptic patients. We have established a scoring scale to assess the state of the animal and detect the first signs of convulsive seizures early and precisely. We demonstrated substantial changes in systemic haemodynamics caused by convulsive seizures. The translational value of this large animal model can be enhanced by comprehensive assessment of cardiovascular, respiratory, and autonomic function during seizures, together with sampling blood and cerebrospinal fluid for biomarkers. Reliable, clinically relevant models of convulsive seizure are essential for preclinical trials of possible prophylactic treatments for SUDEP.

## Data Availability

The data that support the findings of this study are available from the corresponding author upon reasonable request.
